# Supplementary material for: Genetic divergence and phenotypic plasticity contribute to variation in cuticular hydrocarbons in the seaweed fly Coelopa frigida
Source: Ecol Evol. 2019 Oct 2;9(21):12156–70. doi: 10.1002/ece3.5690 (PMC6854331; doi:10.1002/ece3.5690)
Supplement: Supplementary file 1 [file ECE3-9-12156-s001.pdf]

Supplemental Information

Table S1 –GC-MS instrument settings

Injection	1 µl
Inlet	pulsed splitless (1min), 280°C
Column	30m x 0.25 mm I.D. x 0.25µm HP-5MS<br>Max. temperature 325°/350°C<br>Carrier gas: helium<br>Flow: constant flow (0.5 ml/min)<br>Inlet: front inlet<br>Outlet: MSD
GC oven	200°C (3 min), 8°C/min to 325°C, 325°C (10 min)
MSD transfer line	280°C
MS scan	40-600 m/z, 3 min solvent delay
MS temperatures	MS Quad 150°C, MS Source 230°C

Table S2 – Pair-wise tests for sex and population

**Population differences within females**			t-Test			PERMDISP		
			t	p-value		t	p-value	
Ystad	vs.	Stavder	1.240	0.083		0.939	0.462	
Ystad	vs.	Skeie	1.207	0.071		0.015	0.985	
Ystad	vs.	Østhassel	1.261	0.045	*	1.232	0.321	
Stavder	vs.	Skeie	1.654	<0.001	***	1.199	0.313	
Østhassel	vs.	Stavder	1.565	0.002	**	0.467	0.708	
Østhassel	vs.	Skeie	0.916	0.686		1.528	0.212	

**Population differences within males**			t-Test			PERMDISP		
			t	p-value		t	p-value	
Ystad	vs.	Stavder	1.919	<0.001	***	2.316	0.085	
Ystad	vs.	Skeie	1.282	0.041	*	0.102	0.935	
Ystad	vs.	Østhassel	1.146	0.090		0.658	0.594	
Stavder	vs.	Skeie	2.053	<0.001	***	2.545	0.052	
Østhassel	vs.	Stavder	1.855	<0.001	***	2.070	0.087	
Østhassel	vs.	Skeie	1.124	0.167		0.640	0.624	

**Sex differences within populations**			t-Test			PERMDISP		
			t	p-value		t	p-value	
Ystad: F	vs.	M	1.204	0.074		1.039	0.368	
Stavder: F	vs.	M	2.026	<0.001	***	3.841	0.005	**
Skeie: F	vs.	M	1.506	0.007	**	1.514	0.251	

Table S3 – Model significance estimates

Model	No. predictive components	No. orthogonal components	R^2X(cum)	R^2Y(cum)	Q^2(cum)	RMSEE	p R^2Y	p Q^2
**Sex and population effects**								
PCA	8		0.51					
OPLS-DA for sex	1	3	0.36	0.76	0.57	0.25	0.001	0.001
PLS-DA for population	4		0.34	0.5	0.27	0.31	0.001	0.001
OPLS-DA for countries	1	1	0.28	0.52	0.41	0.35	0.002	0.001
**Sex and diet effects in Ystad population**								
PCA	6		0.54					
OPLS-DA for sex	1	3	0.41	0.83	0.5	0.22	0.001	0.001
OPLS-DA for wrack type	1	3	0.44	0.9	0.72	0.17	0.001	0.001

RMSEE = root mean square error of estimation

Table S4 – AIC comparison of models for A. Total peak area, B. Proportion of alkenes, C. Proportion of methylated compounds, D. Weighted mean chain length, E. Dispersion around mean chain length.

A.

Model Terms

Intercept	Country	Sex	Country*Sex	df	Log Liklihood	AIC	Delta
1.712		+		3	-95.545	197.3	0
1.657	+	+		4	-95.117	198.6	1.31
1.71	+	+	+	5	-94.716	200	2.71
1.915				2	-101.032	206.2	8.85
1.86	+			3	-100.647	207.5	10.21

B.

Model Terms

Intercept	Country	Sex	Country*Sex	df	Log Liklihood	AIC	Delta
-1.499	+	+		4	170.256	-332.1	0
-1.544	+	+	+	5	171.11	-331.6	0.5
-1.678		+		3	163.176	-320.1	12
-1.855	+			3	143.044	-279.8	52.26
-2.043				2	137.998	-271.9	60.23

C.

Model Terms

Intercept	Country	Sex	Country*Sex	df	Log Liklihood	AIC	Delta
-0.2605	+	+		4	78.825	-149.2	0
-0.2268	+	+	+	5	79.104	-147.6	1.65
-0.1558		+		3	76.213	-146.2	3.06
-0.09456	+			3	72.483	-138.7	10.52
0.008819				2	70.163	-136.2	13.04

D.

Model Terms

Intercept	Country	Sex	Country*Sex	df	Log Liklihood	AIC	Delta
26.88	+	+		4	-121.059	250.5	0
27.03	+			3	-122.988	252.2	1.69
26.92	+	+	+	5	-120.92	252.5	1.93
27.16		+		3	-127.504	261.2	10.72
27.31				2	-129.211	262.5	12.02

E.

Model Terms

Intercept	Country	Sex	Country*Sex	df	Log Liklihood	AIC	Delta
8.985	+	+		4	-209.006	426.4	0
8.976	+	+	+	5	-209.005	428.6	2.21
8.316		+		3	-215.685	437.6	11.19
10.16	+			3	-227.381	461	34.59
9.492				2	-232.159	468.4	42.02

Table S5 – Type II analysis of deviance tables for best models for: A. Total peak area, B. Proportion of alkenes, C. Proportion of methylated compounds, D. Weighted mean chain length, E. Dispersion around mean chain length.

A.

Term	Df	Chisq	Pr(>Chisq)
Sex	1	11.353	0.0007534

B.

Term	Df	Chisq	Pr(>Chisq)
Country	1	15.002	0.0001074
Sex	1	67.906	< 2.20E -16

C.

Term	Df	Chisq	Pr(>Chisq)
Country	1	5.352	0.020699
Sex	1	13.458	0.000244

D.

Term	Df	Chisq	Pr(>Chisq)
Country	1	13.3253	0.0002618
Sex	1	3.8166	0.0507463

E.

Term	Chisq	Df	Pr(>Chisq)
Country	1	13.841	0.0001989
Sex	1	42.808	6.04E-11
